# Zika: information in the nick of time

**DOI:** 10.7189/jogh.07.010305

**Published:** 2017-06

**Authors:** Jessica L Walker, James H Conway, James E Svenson

**Affiliations:** 1 University of Wisconsin School of Medicine and Public Health, Madison, Wisconsin, USA; 2 Department of Emergency Medicine, University of Wisconsin School of Medicine and Public Health, Madison, Wisconsin, USA; 3 Department of Pediatrics, University of Wisconsin School of Medicine and Public Health, Madison, Wisconsin, USA

The association of Zika virus with microcephaly and other neurologic issues has led to widespread concern throughout the world. These concerns have led local and national government health agencies to take a variety of measures to inform the public, and to prevent the spread of the virus. In Florida, for example, there was widespread spraying in a Miami neighborhood where 16 cases of autochthonous transmission had been found, and an urgent travel alert issued quickly [1]. Newspapers rapidly began urging people, especially pregnant women, to take specific precautions. They offered suggestions that included chemical clothing treatment, wearing long sleeves and pants, and even staying indoors as much as possible [2]. In February 2016, CBS reported that mosquito repellent sales in the United States had already increased by 11.9% from 2015 [3].

Some of the success of government efforts lies in the perceived threat of the virus. In Puerto Rico, despite a widespread information campaign, there was clearly resistance to mosquito control efforts, and the incidence of cases increased steadily [4]. To be most effective, public health campaigns must provide information to the people and help them understand the threat of the disease, in language and format that are accessible to that population. To further understand the “knowledge gap,” we conducted an informal survey of knowledge of Zika and the potential consequences in an endemic area of rural Guatemala.

**Figure:**
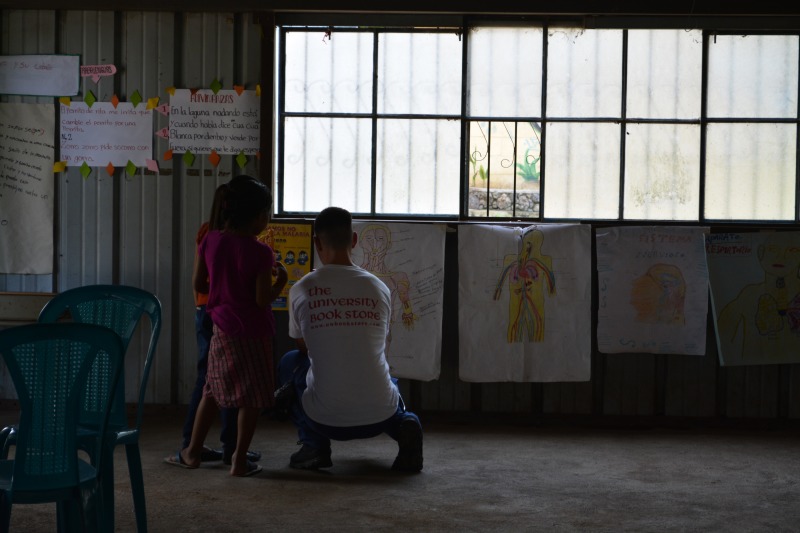
Photo: How children are learners and potential teachers (from Jessica Walker’s personal collection)

For two weeks in May and June of 2016, a group of medical students and physicians from the University of Wisconsin School of Medicine and Public health worked in ten different rural villages outside of San Lucas Tolíman, Guatemala, as part of an annual collaborative outreach project. The primary intent for this program is to provide basic medical care to populations with poor health care access, as well as basic preventive health education, but the clinics also provided an opportunity to assess baseline knowledge about Zika in local populations. At their clinic intake, each patient was queried regarding what they knew about mosquito–borne illnesses. If they did not mention Zika, they were asked if they had heard of the disease. Finally, patients were asked what they do to protect themselves and their families from mosquitos.

A total of 116 patients were surveyed. 46% of people had heard of Zika, 39% of people had heard of Chikungunya, 40% of Dengue, and 25% had no knowledge of any mosquito–borne illnesses. The results were not uniform across villages, but patients generally had little knowledge about the symptoms and consequences of Zika. For example, some surmised that Zika caused nosebleeds, while this would more likely be a symptom associated with severe dengue infection. In one village, nearly all patients reported that they burn garbage to get rid of mosquitos, a practice that may not be useful and certainly could have other detrimental health effects. Patients seemed much better informed about diseases that had recently plagued their villages, such as chikungunya, which reached epidemic proportions in 2015 [5].

So why did this rural population have such little knowledge about the current threat of Zika? One answer may lie in the more detailed knowledge of chikungunya and dengue, diseases that have affected, and still do affect, these communities. It stands to reason that patients’ ability to better describe these diseases may be from having experienced them already, as these diseases have circulated in the area for many years. However, if we make that assumption, it raises another troubling issue; the percentage of patients who were able to identify these endemic diseases was still less than 50%. This figure suggests that, even in areas affected by dengue and chikungunya, education about recognition and prevention has been suboptimal. When considering a disease as dangerous as dengue, which is estimated to hospitalize 500 000 people each year and kill at least 12 500, the knowledge gap surrounding mosquito–borne illness must be addressed [6].

So what can be done in resource–limited areas to improve knowledge and limit mosquito–borne disease? At the level of public infrastructure, the government in Guatemala and organizations such as the Red Cross are increasing spraying efforts and distributing insect repellent in order to limit mosquito bites in response to Zika [7,8]. On the public information side, our survey yielded some positives, as some patients did have accurate information about the threat of Zika. Their sources of information were community health promoters and primary schools, where children educated about the virus brought information home to their families. These sources of information are readily accessible vehicles for public health education efforts. Perhaps using Zika as a newly emerging threat could offer a new opportunity to share the importance of all vector–borne infections, open new avenues of communication, and foster the development for more comprehensive education on all severe mosquito–borne illnesses. If we start by educating children and community health promoters, the information may be disseminated more quickly within the community.

The public’s captivation with Zika is likely to wane over time, but the reality is that other diseases such as yellow fever, malaria, dengue and chikungunya are endemic in many regions, and control remains elusive. The successful dissemination of information in areas where technology may be limited is imperative, to help limit the impact of the mosquito epidemic in Latin America and other resource limited settings. It is not enough to provide mosquito repellent and bed nets, or to spray insecticide, if the people do not understand the importance of these interventions. Many communities remain afflicted by these illnesses because they are unaware of the mechanisms of transmission or effective personal protection interventions.

Coordinated efforts to educate, support, and follow up with these communities is needed, in order to ensure that there is genuine understanding, as well as a sense of empowerment against these diseases. Word of mouth travels rapidly in countries with little access to technology, and as evidenced by our experience, schools can be used as a valuable educational and informational resource. Every opportunity must be found to utilize public health and educational activities, in coordination with governmental and private sector interventions.

As it stands now, there is no cure for most of these diseases, but there is prevention. And in the future, there may be more an array of vaccines to offer for many of these conditions, but acceptance will partially depend on understanding of the importance of these diseases. We must seize the opportunity that Zika has provided and re–start the conversation in these areas. Vector–borne diseases do not have to be considered inevitable.
